# Dynamic monitoring of vital functions and tissue re-organization in *Saturnia pavonia* (Lepidoptera, Saturniidae) during final metamorphosis by non-invasive MRI

**DOI:** 10.1038/s41598-022-05092-3

**Published:** 2022-01-20

**Authors:** Tim Laussmann, Paul Urspruch, Vera Flocke, Anton G. Windfelder, Hermann Aberle, Klaus Lunau, Ulrich Flögel

**Affiliations:** 1 Scientific Association Wuppertal e.V., Gierener Weg 19, 51379 Leverkusen, Germany; 2 Experimental Cardiovascular Imaging, Department of Molecular Cardiology, Heinrich Heine University, Universitätsstraße 1, 40225 Düsseldorf, Germany; 3 Branch for Bioresources, Fraunhofer Institute for Molecular Biology and Applied Ecology IME, Ohlebergsweg 12, 35392 Giessen, Germany; 4 Institute of Functional Cell Morphology, Heinrich Heine University, Universitätsstraße 1, 40225 Düsseldorf, Germany; 5 Institute of Sensory Ecology, Heinrich Heine University, Universitätsstraße 1, 40225 Düsseldorf, Germany; 6 Cardiovascular Research Institute Düsseldorf (CARID), Heinrich Heine University, Universitätsstraße 1, 40225 Düsseldorf, Germany

**Keywords:** Developmental biology, Imaging techniques

## Abstract

Magnetic resonance imaging (MRI) is the key whole-body imaging technology for observing processes within a living object providing excellent resolution and contrast between soft tissues. In the present work, we exploited the non-destructive properties of MRI to track longitudinally the dynamic changes that take place in developing pupae of the Emperor Moth (*Saturnia pavonia*) during the last days before eclosion. While in diapause pupae, body fluid was almost homogeneously distributed over the internal compartments, as soon as wings, legs, flight muscles and the head region were fully developed, a significant redistribution of water levels occurred between thoracic and abdominal regions. During the last two days before eclosion, the developing moths transferred substantial amounts of liquid into the gut and the labial gland, and in case of females, into developing eggs. Concomitantly, the volume of the air sacs increased drastically and their expansion/compression became clearly visible in time-resolved MR images. Furthermore, besides ventilation of the tracheal system, air sacs are likely to serve as volume reservoir for liquid transfer during development of the moths inside their pupal case. In parallel, we were able to monitor noninvasively lipid consumption, cardiac activity and haemolymph circulation during final metamorphosis.

## Introduction

The Emperor Moth (*Saturnia pavonia*) a member of the Saturniidae family is quite common in the Palaearctic area, occupying moorland and open country. In contrast to the hawk moth *Manduca sexta*, which commonly serves as a model organism^1^, *Saturnia pavonia* imagoes do not feed. Thus, the moths mate immediately after eclosion in early spring after hibernation. Males are persevering flyers and usually active in the afternoon and follow pheromone traces produced by females. They are capable of finding females over several kilometres^2–4^. The abdomen of the females is already filled with about 200 fully developed eggs, which are laid directly after mating, often during the following night. Usually, females are quite lethargic and place their eggs around twigs of *Prunus, Salix,* and *Crataegus,* and many other plant species in close surroundings. Caterpillars develop in early summer and prepare a solid silk cocoon with integrated calcium oxalate hydrate crystals^5^ for the hibernating obtect pupa. The cocoon has a prepared opening acting as a gate for the hatching moth. On the surface of the pupa, the sheaths of the thoracic appendages are fixed to the pupal body^6^. Thus, pupae are only capable of moving the hindmost part of the abdomen. Female pupae have twice the mass compared to male pupae^4,7^. Low winter temperatures trigger termination of the diapause^8^, but with varying susceptibility between individuals. Some pupae do not release the moth after the first hibernation. In fact, *Saturnia* pupae are able to hibernate at least up to five winters^9^. Most likely, these “over-lying” pupae are in a state of diapause or arrested development, which ensures survival of offspring during years with adverse environmental conditions. Hatching moths spit a slightly alkaline liquid, which is needed as a lubricant to pass through the prepared cocoon opening^10^. In species with completely closed cocoons, this liquid, originating from the labial glands, contains a proteolytic enzyme known as cocoonase^11,12^. This enzyme is capable of digesting sericin, a protein glue between the silk fibers of the cocoon^12^. However, this enzyme is absent in *Saturnia pavonia*^10^.

Although the metamorphosis of moths and butterflies is common textbook knowledge, the development in the pupal stage remains enigmatic due to methodological shortcomings. In recent years, technological improvements especially in X-ray micro-computed tomography (micro-CT) have led to a number of studies on insect metamorphosis, especially in flies^13^. With this technique internal morphological alterations can be visualized at very high-resolution^14–17^ and by time resolved X-ray imaging also dynamic processes, such as movement of air trapped in an “air bubble” from inside the pupae to the space between the adult insect and the puparium^18^. Modern micro-CT systems can yield virtual sections of the insect brain and the optical lobe^19,20^ and even visualise infectious nematodes in its vector, black flies^21^.

In contrast to micro-CT, magnetic resonance imaging (MRI) provides superior contrast between soft tissues and allows a quantitative determination of water and fat content. With this technique, water and fat distribution have been determined in lepidopteran pupae (*Pieris brassicae* and *Graphiphora augur*)^22^ as well as lipid consumption of a beetle after flight^23^. Furthermore, MRI has been applied to visualize alterations in the respiratory, circulatory and alimentary system as well as cerebral and alimentary tract development of *Manduca sexta*^24–26^. Additionally, Mapelli et al.^27^ studied a single specimen of *Bombyx mori* during the major steps of its life cycle, from young larva to moth. However, up to now no developmental MRI investigations have been carried out in *Saturnia pavonia*.

Thus, in the present work, we visualised and studied the processes that take place within male and female pupa during the last 6 days before the moth hatches using high resolution MRI. We demonstrate day-to-day shifts in tissue properties of the different organs within the developing pupae and resolve dynamic alterations caused by air sac ventilation and cardiac activity during the late phase of metamorphosis. Given the substantial differences in life history, especially the immediate egg development and mating strategy of *Saturnia pavonia*, the present study about its final metamorphosis complements the understanding for development of different moth pupae species.

## Results

### Morphological features

**Figure 1:**
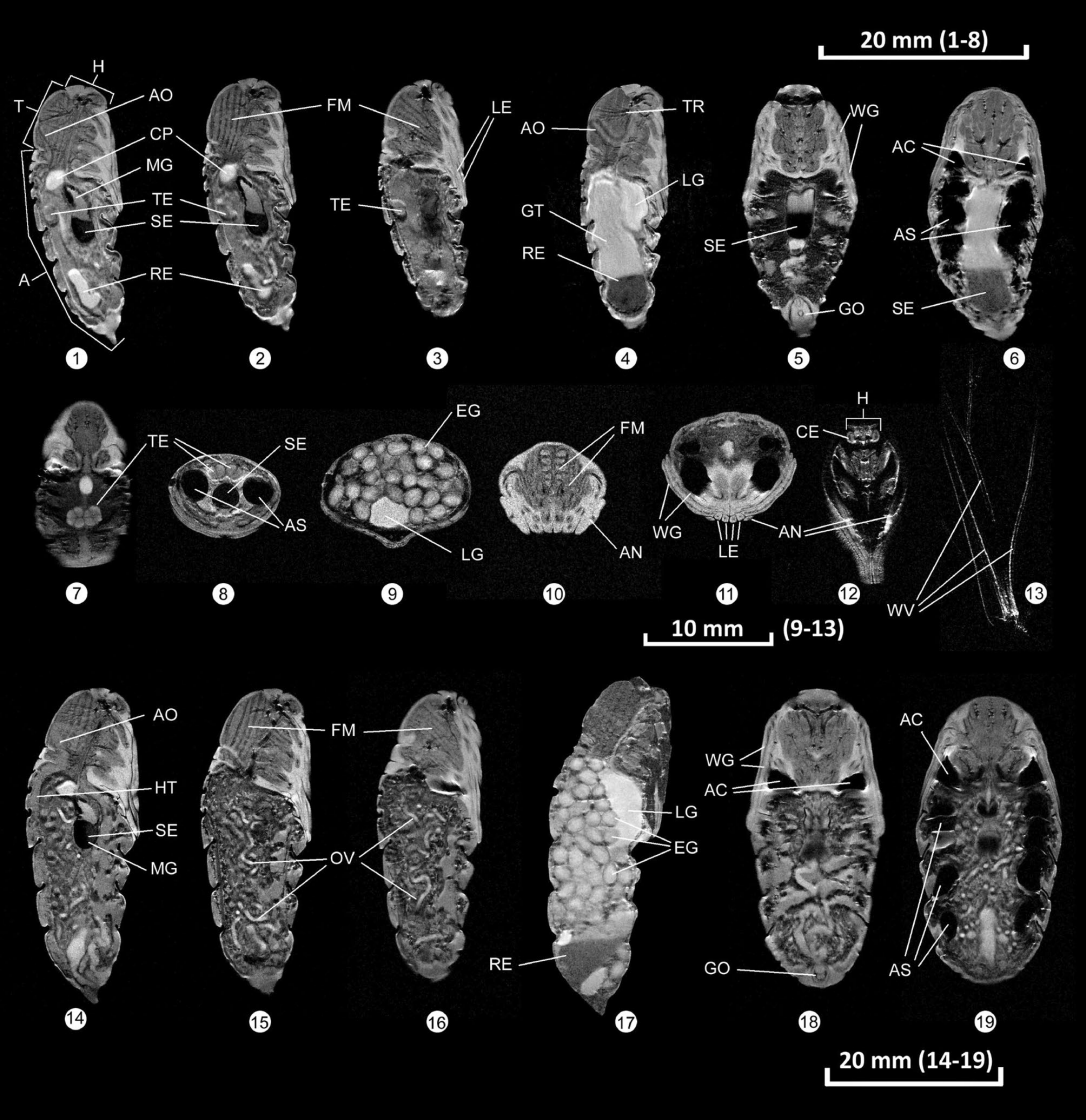
Morphological features of pupal development in *Saturnia pavonia*. First row (1–6): Male pupae at different developmental stages (1–4: sagittal view, 5 and 6: coronal view). Second row (7–13): Selected, slightly enlarged views of interest from males and females (7 and 12: coronal view, 8–11 axial view, 13: fully developed wing of an imago immediately after eclosion). Third row (14–19): Female pupae at different developmental stages (14–17: sagittal view, 18 and 19: coronal view). A abdomen, AO aorta, AC air cavity, AN antenna, AS air sacs, CE compound eye, CP crop, EG egg, FM flight muscle (dorsoventral and dorsolateral), GO genital opening, GT gut, H head, HT heart tube, LE leg, LG labial gland, MG midgut (filled with aqueous liquid (bright) and a sediment (dark)), OV ovaries, RE rectum, SE sediment, T thorax, TE testicle, TR trachea, WG wing and wing sheaths, WV wing veins.

At an in-plane resolution of ~ 30 × 30 µm^2^, both major organs and tiny structures of developing *Saturnia pavonia* pupae became clearly visible by non-invasive MRI. Of note, the pulse sequence used for data acquisition resulted in T2-weighted images emphasizing tissues with high water and/or fat content as bright structures. Scanning of the pupae over time (n = 5 each sex) revealed that different morphological features were most pronounced in specific developmental stages. Therefore, we provide a collection of images illustrating the evolution of distinct features over the entire observation period in Fig. 1. The first row, images 1–3, shows three different slices in sagittal orientation of a male pupa 5 days before eclosion (dorsal: left, ventral: right), while images 5 and 6 provide two different coronal sections of the same pupa. In between, image 4 offers a sagittal view of the pupa two days before eclosion. Similarly, a female pupa is depicted in the third row of Fig. 1 (images 14 to 19). Furthermore, the second row shows selected views focusing on distinct properties of interest. As can be recognized, the U-shaped aorta (AO) centrally located in the thorax (T) became clearly visible in images 1 and 4. As a salient structure in the thorax (T), the flight apparatus (FM) consisting of longitudinal and transversal muscles could be resolved in images 2, 3 and 10. In the abdomen (A), the crop (CP) filled with aqueous liquid appeared as a bright spot. The testes (TE) were located dorsally in the middle of the abdomen of males and could be clearly seen in images 7 (coronal view) and 8 (axial view) as a pair of “four-leaf clover” structures.

Another prominent feature in pupae was the midgut (MG) filled with sediment (SE) and residual water (Fig. 1; images 1, 2 and 5). Interestingly, when turning the pupa by 180 degrees, this sediment—which most likely consists of insoluble metabolic end products, e.g. uric acid and allantoin^28^—followed gravity (Supplemental Fig. 1). In the early phase of metamorphosis, the rectum (RE) was filled with an aqueous liquid. The legs (LE) were located ventrally in sheaths filled with liquid (Fig. 1; images 3 and 11). Similarly, the sheaths of the wings (WG) and antennae (AN) were closely attached to the thorax (Fig. 1; images 5, 10, 11 and 12). In female pupae (images 14–19) the most striking features were the ovaries (OV) in the abdomen (A). Of note, two days before eclosion, the eggs (EG) were fully developed (Fig. 1; images 9 and 17). At the same time, the labial glands (LG) cranial-ventral located in the abdomen and filled with liquid became visible (Fig. 1; images 4, 9 and 17). In coronal views, the genital opening (GO) could be visualized (Fig. 1; images 5, male and 18, female). The head (H) with its compound eyes is (CE) fully developed two days before eclosion as can be recognized in a more proximal slice (Fig. 1; image 12). Between abdomen, thorax and wings appeared an air cavity (AC) in developing pupae (Fig. 1; image 6). Already early in metamorphosis, organs with dynamic functions became visible, the dorsal heart tube (HT, image 14) and the air sacs (AS, Fig. 1; images 6, 8 und 19), located laterally in the abdomen. Please note, after eclosion and wing inflation, the tube-in-tube structure of the wing veins (WV) could be clearly resolved in the imago (Fig. 1; image 13) with the inner tube (trachea) filled with air (dark area in the center of veins) and the outer filled with hemolymph (bright linear areas).

### Developmental changes

**Figure 2:**
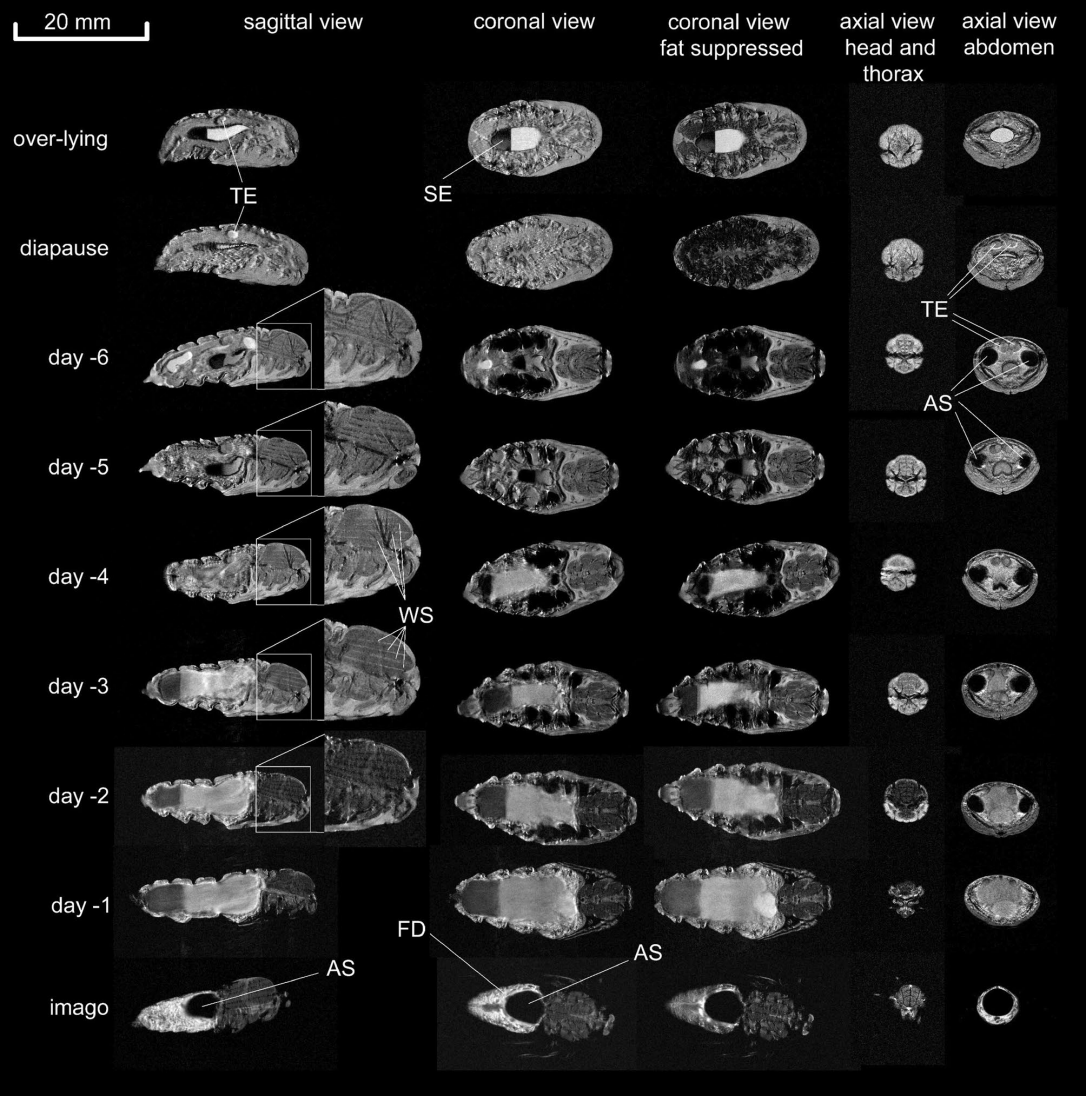
Development of a male pupa from diapause to imago on a day-to-day basis. Sagittal and axial view of the abdomen of male pupae showing the anlages of the testes (TE) in the diapause. Before the moth withdrew the aqueous liquid from the leg and wing sheaths, lines with intense water signal (WS) appeared between the flight muscles. Air sacs (AS) were deflated during diapause and evolved during development (see axial view of the abdomen). Shortly before eclosion, air sacs are marginalised by the inflating gut and rectum filled with meconium consisting of soluble and insoluble metabolites. In male moths, a residual fat deposit (FD) around the gut is evident when comparing non-suppressed and fat-suppressed images. In imagoes, air sacs move to a central position in the abdomen close to the metathorax; SE sediment.

**Figure 3:**
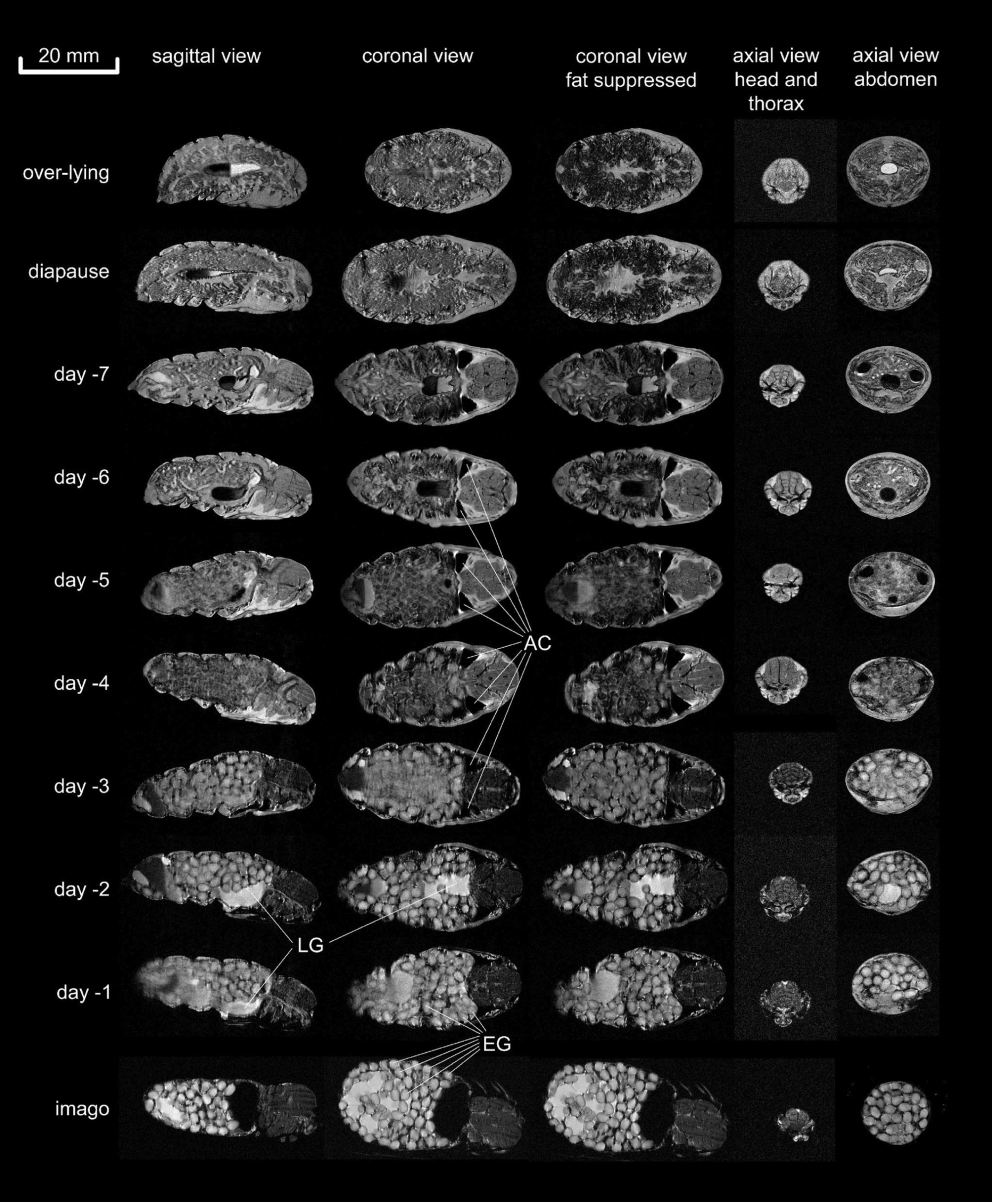
Development of a female pupa from diapause to imago on a day-to-day basis. While many processes were similar in female and male pupae, the most striking development in the female body was the massive production of eggs (EG) which filled the female’s abdomen almost completely. The only other prominent abdominal features are the labial glands (LG) appearing as a bright cranial-ventral object and the gut filled with water and sediment. In female imagoes, a liquid reservoir is visible close to the genital opening, most likely containing the “glue” that is needed to fix the eggs on the substrate during oviposition; AC air cavity.

The development of male and female pupae during metamorphosis is illustrated on a day-to-day basis in Figs. 2, 3, respectively. Column 1 shows in each case a medial slice in sagittal view, while columns 2 and 3 depict medial slices in coronal view without (column 2) and with (column 3) fat suppression—thus, areas that occur darker in column 3 *vs.* 2 exhibit significant amounts of fat depots (triglycerides). Axial views of head and thorax as well as abdomen are given in columns 4 + 5, respectively. As a kind of baseline controls, the upper two rows show an over-lying pupa which did not hatch after the first winter (first row) and a diapause pupa before the first winter (second row), which successfully eclosed thereafter. In the following rows, examples of the development of pupae are illustrated from day 6 to 1 (male) or day 7 to 1 (female) before eclosion with the final images of the imagoes in the last row.

In both types of resting pupae (over-lying and diapause, Figs. 2, 3, first and second row) no detailed structures were visible. However, compartments like head, thorax, and abdomen, as well as the wing, legs and antennae sheaths were already established and can be clearly recognized. On the other hand, the midgut filled with liquid and sediment was variable in size in different pupae. Another pronounced structure in diapause pupae were the anlages of the testes (TE) in males and ovaries in females. Of note, the air sacs seemed to be deflated during diapause and were not visible. However, six days before eclosion, male and female pupae already showed inflated air sacs (AS). Thus, as soon as metamorphosis is triggered by low temperatures in winter, the activated pupae fill their air sacs with air to supply the required oxygen for the increasing energy demand in the final phase of development. In line with this observation, at the same time tissue fat levels started to decline (see below). Interestingly, we noticed very different air sac volumes within the same animals in day-to-day measurements, indicating that there is a low-frequency tidal flow of air in and out of the air sacs. Actually, a higher ventilation rate would have led to motion artefacts in the images due to data acquisition time over ~ 4 min. This observation encouraged us to monitor this process in time-resolved images (see section *dynamic features*). Besides the air sacs, an air-filled cavity (AC) between abdomen, thorax and wings developed. During the last six days, the volume of the midgut increased continuously, as did the volumes of the hindgut and rectum. From this we infer that during this time, fluid and metabolites were secreted into the midgut and transferred to the hindgut and rectum. This was accompanied by a deflation of the air sacs immediately prior to eclosion.

Flight muscles (FM), eyes, legs, and wings developed early after metamorphosis was initiated. On day six before eclosion, the first intimations of flight muscles (FM) were already visible. Of note, during that period of development, the water content in the head and thorax region was much higher than at later time points. Here, the wings, antennae and leg sheaths were filled with an aqueous liquid. Three days later, all structures and organs in the thorax and head region seemed to be fully developed. Interestingly, at this time, the developing moths obviously withdrew water from the head, thorax and appendage sheaths, which was associated with an intercalation of water (WS) between the flight muscles (Fig. 2, sagittal view, enlarged views of the thorax). This process is further scrutinized below (see section water and fat distribution during metamorphosis). In parallel, the testes developed in male pupae and shifted from a dorsal to a dorsolateral position (Fig. 2, fifth column). In the abdomen of females, the ovaries were already developing 7 days before eclosion. First intimations of eggs became evident on day 5 before eclosion. In the following days, the eggs developed rapidly and the abdomen was almost completely filled with eggs in pharate adult females (i.e. the stage at which the adult insect body appears to be fully developed inside the pupal cuticle). In male pupae, the rectum was further filled with residual liquid. Another pronounced structure became visible two days before eclosion: At this time the labial glands (LG) were filled with an aqueous liquid. When comparing coronal images with and without fat suppression (Figs. 2, 3, columns 2 + 3), it became obvious that the diapause pupae contained a large amount of diffuse fat deposits around gut and midgut, which was consumed during the further development. However, some of these fat deposits (FD) were still visible in the fully developed male before and after eclosion. We describe the alterations in fat content of pupae during metamorphosis in more detail in the section water and fat distribution during metamorphosis. Of note, in the supplement we provide the entire dataset of images in high resolution as plates for poster print.

### Dynamic features in developing pupae

**Figure 4:**
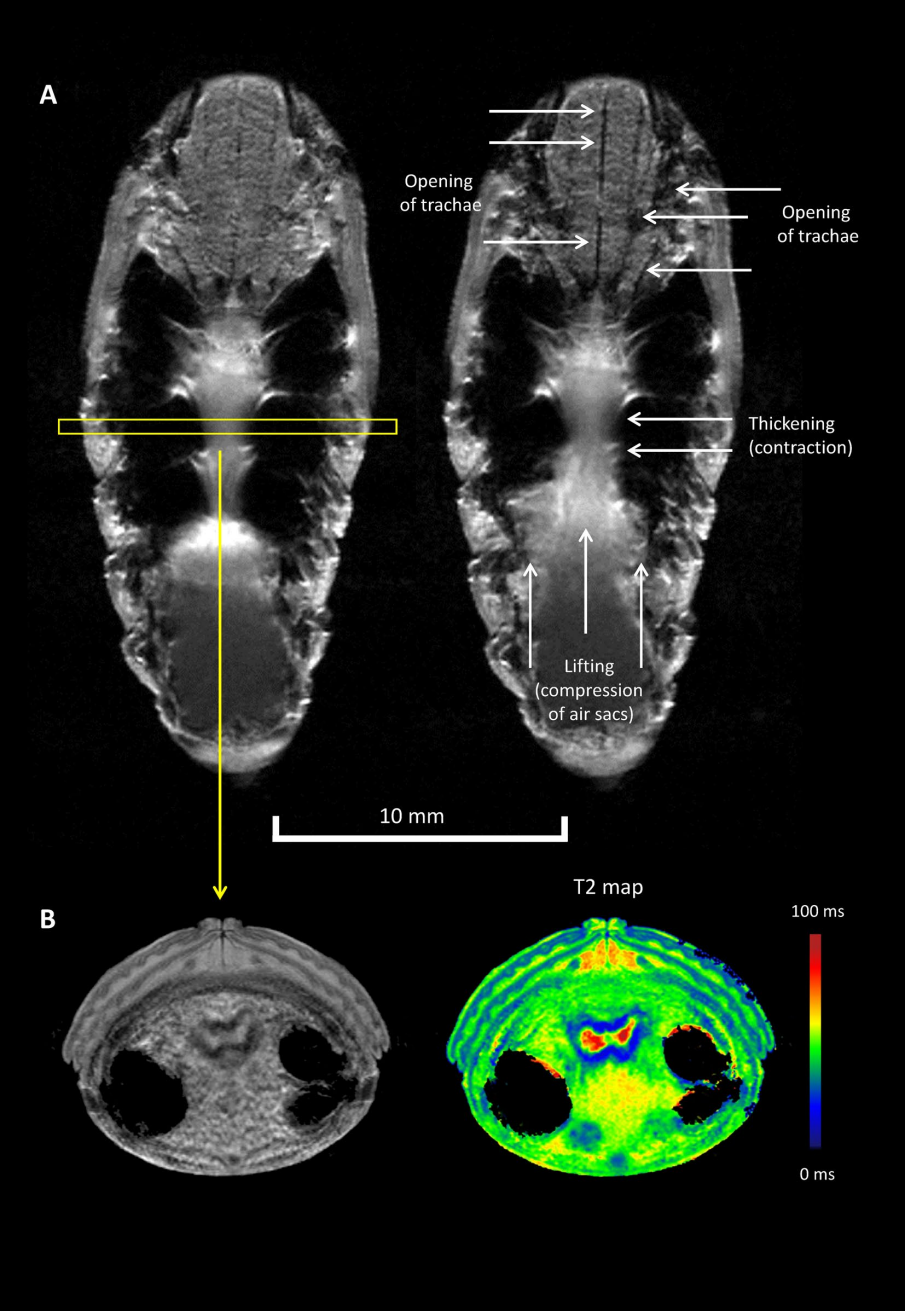
Visualization of active ventilation. (**A**) Sequential coronal sections illustrate air sac compression by contraction and lifting of the abdominal tract leading to opening of tracheae allowing active ventilation. The yellow rectangle indicates the imaging plane in (**B**). (**B**) Axial sections relating anatomy and MR relaxometry indicating typical muscle T2 relaxation times around the contracting gut (colour-encoded in blue).

As pointed out above, during measurement of developing pupae on a day-to day basis it became evident that the air sacs were quite variable in volume. To explore whether this was caused by active ventilation, we decided to acquire time-resolved images of the pupae, which indeed revealed ventilatory activity with a rate of 10–20 h^−1^, which remained almost constant when pupae were continously imaged overnight (data not shown). Strikingly, this was accompanied by caudal air sac compression and at the same time by tracheal dilation in the thorax (Fig. 4A and Supplemental Movie 1 (male, coronal orientation, 60 frames over 1 h)). T2 maps indicated that the gut is surrounded by muscular tissue—usually characterized by a T2 of ~ 25 ms at 9.4 T (colour-encoded in blue; Fig. 4B)—which seems not only responsible for abdominal motility but also for ventilation. Of note, the observed internal air sac compression was not accompanied by an external contraction of the abdomen (cf. Supplemental Movies). Thus, an indirect compression of the air sacs by external transversal or longitudinal compression of the abdomen can be excluded.

**Figure 5:**
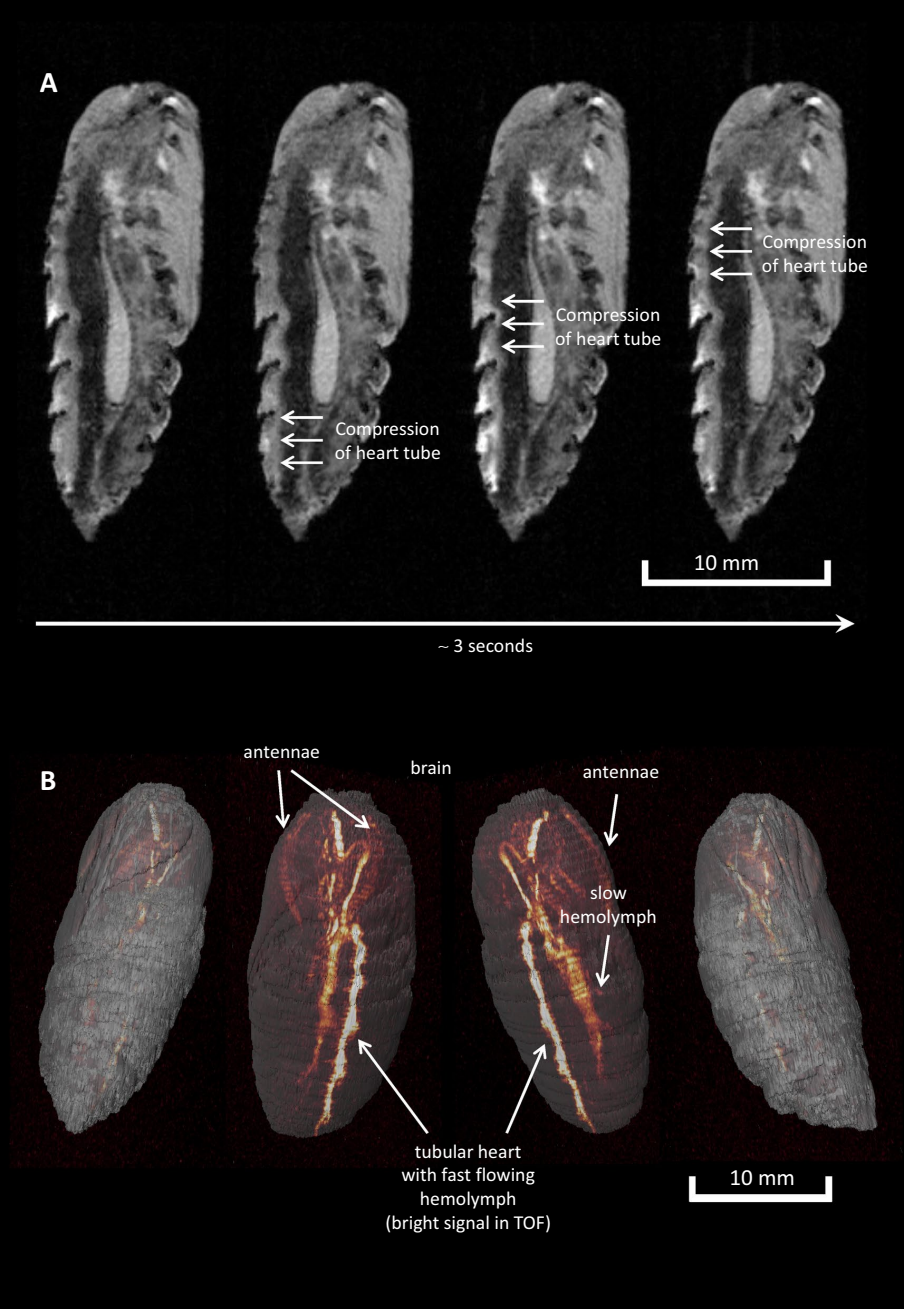
Cardiac contraction and flowing hemolymph. (**A**) Sequential sagittal sections showing successive compression of the heart tube. (**B**) Different views on 3D reconstructed time-of-flight (TOF) MR angiography data illustrating the path of the flowing hemolymph within pupae of *S. pavonia*.

Using self-gated cine sequences, at the same time pumping activity of the dorsal heart tube could be visualized without motion artefacts (Fig. 5A and Supplemental Movie 2, heart rate ~ 20 min^−1^ (male, sagittal orientation, 16 frames over 3 s)). Finally, time-of-flight MR angiography (TOF MRA) was successfully applied to image the flowing haemolymph in the pupal body (Fig. 5B, Supplemental Movie 3): Active pumping causes a bright signal in the tubular heart due to the fast flowing blood, while the passive back flow of the hemolymph is much slower leading to weaker signals in TOF MRA. Interestingly, also the perfusion of the antenna became clearly visible. Time-resolved imaging of female pupae revealed furthermore pronounced egg movement within the ovaries (Supplemental Movie 4 (240 frames over 2 h)). All dynamic features observed in developing pupae are compiled in Supplemental Movie 5 together with some explanatory notes.

### Water and fat distribution during metamorphosis

**Figure 6:**
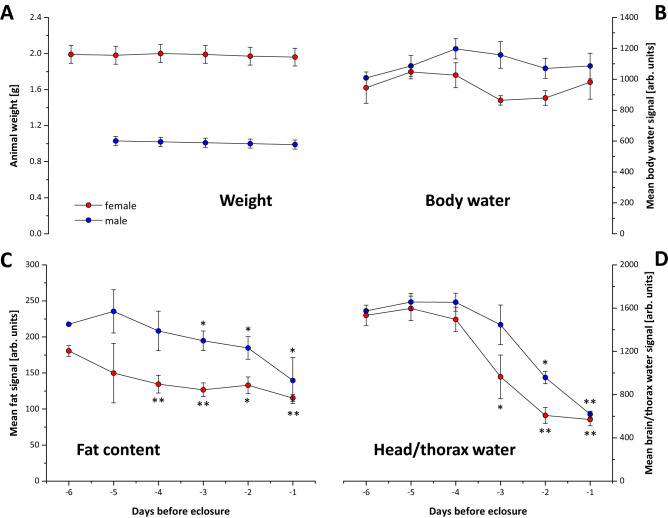
Temporal development of weight, water and fat content of pupae over the observation period. (**A**) weight, (**B**) mean total water signal, (**C**) mean fat signal and (**D**) mean brain/thorax water signal. Data are means ± SD; * = *P* < 0.05, ** = *P* < 0.01 *vs.* day -6 (n=5 each).

The total weight of female pupae was two-fold higher than that of male pupae and did not change significantly in both sexes during development (Fig. 6A). Both male and female pupae also exhibited a balanced total water content during the last days before eclosion (Fig. 6B). However, as already indicated above, the regional water signal intensity in the head/thorax region dropped by more than 50% between days 4 and 2 before eclosion (Fig. 6D). In particular, the water from the appendage sheaths was withdrawn and replaced by air while development of the head and thorax segments was accomplished (Figs. 2, 3). In parallel, over the entire observation period, the fat content decreased by ~ 40% in both female and male pupae (Fig. 6C).

## Discussion

In the present study we employed high resolution MRI to monitor the final metamorphosis in female and male pupae of *Saturnia pavonia*. With this, even tiny structures within *Saturnia pavonia* pupae became clearly visible and we were able to follow the development of both sexes in detail. Fat/water separation allowed us to characterize substantial liquid redistribution and lipid consumption during the final days before eclosion. Furthermore, time-resolved imaging could visualize dynamic processes inside the living pupae, such as active ventilation, cardiac activity and egg motion, which to the best of our knowledge for insects have not been reported before in the presented quality.

### Development and liquid redistribution

Since both sexes of the Emperor Moth cannot feed as imagoes and mating takes place soon after eclosion, the development of the reproductive organs and the eggs have to be finished as soon as the moths hatch from their pupal case. The indirect flight muscle apparatus consisting of dorsoventral and dorsolateral muscles dominates the thorax^6^. It is already present in the embryo^29^ and presumably develops after diapause from fixed progenitor cells during metamorphosis. MRI could provide non-invasive insight into the processes inside the developing pupae: As soon as the head and thorax region is fully developed, the moth withdraws water from the thorax and the extremity sheaths. Especially, the aqueous liquid located in the thoracic appendages sheaths is rapidly resorbed shortly before eclosion. Prior to this process, a significant water deposition could be detected between the flight muscles. However, the origin of this “intramuscular water” remains elusive. Ligature experiments with *Manduca sexta* pupae revealed that the liquid between the pupal cuticle in the pharate adult is resorbed by both the front and rear openings of the gut^30^. In parallel, the volume of the resorbed liquid is replaced by air. The function and mechanism of this rapid transfer of liquid and air is not yet understood but is probably achieved by a complex interplay of hydraulic and pneumatic activity within the insect body^31^. Most likely, air is transferred from the air sacs (which deflate at the end of metamorphosis) to the appendages sheaths by the tracheal system. In flies it was assumed, that the air between the pharate adult and the puparium might originate from an internal air bubble^32^ (see also next section) as indicated by time-resolved X-ray imaging^18^. The withdrawal of liquid from the appendages sheaths can also be recognized in a video of surgically exposed hindwings from developing pupae of the nymphalid butterfly *Junonia orithya* supplied by Iwata et al.^33^. Subsequently, eggs in the female body inflate rapidly during the last three days before eclosion. The concomitantly observed liquid redistribution from head/thorax to the abdominal egg depot most likely reflects the yolk transfer into egg cells leading to a steady growth of the eggs^6^. Additionally, the labial glands fill with liquid. Since a water-containing connection between the labial glands and the mouth opening is clearly visible (e.g. Figure 3, sagittal view, day -2), it is tempting to speculate—even due to its known similar chemical composition^34^—that the labial glands contain resorbed moulting fluid. The moths will need this liquid as a lubricant for hatching through the cocoon opening^10^.

### Air sacs and ventilation of the tracheal system

During diapause, the air sacs are deflated but as soon as development starts, the internal volume of the air sacs increases. Of note, also during pupal development of the nymphalid butterfly *Vanessa cardui* the tracheal system has been reported to increase dramatically in volume^35^. Most likely, this initial process is associated with an expansion of the abdominal segments. Time-resolved images of the present study demonstrate their ventilatory activity at a frequency of 10–20 h^−1^. Of note, this was not accompanied with any movement of the outer surface of the pupae, indicating that this is a completely internal activity. Similarly, during air sac contraction in *Manduca sexta* no movement of the pupal surface was observed as well^24^. Nevertheless, it remains an open question, whether or not the observed ventilation process implies an active “inhalation” of ambient air. Two days before eclosion, the air sacs deflate again. Thus, it can be assumed that the air sacs serve as volume reservoir during metamorphosis, when liquids are translocated in the pupal case. Interestingly, Hall et al. visualised an air reservoir (“bubble”) in a developing blow fly puparium that is connected to the tracheal system and showed the movement of air from inside the pupae to the space between the pharate adult and the puparium^18^. In this context, it has been suggested that the development of the gas bubble in the abdominal region keeps the body volume constant within the puparium during histolysis^36^. Additionally, released air from bubbles might aid the eversion of head, legs and wings by creating space between the pupa and the puparium^32^.

The almost constant ventilation rate observed in overnight measurements indicates an only minor role of discontinuous gas exchange^37^ in this energy-demanding phase of development. Interestingly, in the already mentioned video of *Junonia orithya* by Iwata et al.^33^, there is also a regular “pulsing” of the exposed wing sheaths visible (with 10–20 pulses per hour) supporting our finding of air sac compression as driving force for this phenomenon. In this context, air sac contraction has been suggested as causative of hemolymph movement^24^ as deduced from an MRI study of pupae from *Manduca sexta*. However, this seems somehow overinterpreted since we found quite different contraction rates for air sacs (10–20 h^−1^) and the dorsal vessel (~ 20 min^−1^) clearly indicating an independent control of these processes and only a supporting function of air sac contraction for hemolymph circulation. Importantly, the air sacs contribute also to a reduction of body density facilitating the flight of adult moths^6^. In the present study, we also observed prominent air sacs in adult *Saturnia pavonia* moths, which were centrally located in the abdomen close to the metathorax, most likely serving as air reservoirs for ventilation of the flight apparatus by compression of the abdominal segments.

Besides the air sacs, an air-filled cavity between abdomen, thorax and wings developed early in metamorphosis. A similar cavity has already been described in other lepidopterans^35,38,39^. Interestingly, the cavity is much smaller in diapause *Pieris rapae* pupae compared to non-diapause pupae. Thus, it was possible to discriminate diapause from non-diapause pupae just by their buoyancy in water^38^. However, this cavity is utterly absent in diapause pupae of *Saturnia pavonia—*it appears when diapause has been terminated, but it remained elusive whether the air is supplied by the tracheal system or just enters from outside the pupal cuticle. Most likely, this cavity is just a free space between abdomen and thorax, covered by wings.

### Energetic considerations

The results of this study indicate that about 40% of the fat storage is consumed during the last 5–6 days of metamorphosis which is in accordance with previous findings^40^. It is particularly noteworthy that a residual fat deposit is present in male imagoes. Actually, the male adult moths are much more active and have to search for females by means of pheromones. After mating, females usually do not fly long distances, but lay their eggs in the immediate vicinity of their site of eclosion. Therefore, it makes sense that males have larger fat deposits in order to supply energy for female seeking and mating^41,42^. In contrast to the fat content, the water content remains equilibrated during metamorphosis. However, as soon as the moth hatches and the wings are inflated it excretes large amounts of meconium (liquid as well as soluble and insoluble metabolites), which leads to significant weight loss in imagoes. Finally, our data show that pupae, which do not hatch after the first winter, stay in a permanent diapause stage without noticeable differences in the water and fat signals of distinct organs as long as environmental conditions (temperature and day-length^8^) do not trigger the metamorphosis process.

## Conclusions

In summary, the current study provides new insights into one of nature's greatest mysteries and phenomena—the fascinating process of metamorphosis what normally takes place hidden and unobserved inside the butterfly pupae. This will not only allow a deeper understanding of insect physiology but our data may also serve as a kind of reference atlas for the final development of moths to assess subtle morphological and functional impairments due to environmental changes, such as pollution or climate, which may help to resolve the underlying pathophysiological mechanisms of insect mortality.

## Methods

### Animals

*Saturnia pavonia* pupae are descended from a fertilised female caught in 2004 in Wuppertal, Germany. In the following years, we continuously mated females of this livestock with free males in Leverkusen, Germany. Caterpillars fed on *Crataegus* species in early spring, later on *Prunus spinosa* and *Salix caprea*. Pupae hibernated above a bowl filled with water in a pupa box at ambient temperature. For the MRI experiments, we transferred the pupae to room temperature in late winter in order to accelerate development of the moth. For better image quality, we removed the cocoon from the pupae. During the course of experiments, we moistened the pupae every day by means of a water sprayer in order to avoid artificial water loss caused by the energy input during the MRI measurements. All animals easily tolerated the applied MRI protocol and developed normally until hatch.

### Magnetic resonance imaging

#### General

Data were recorded at vertical Bruker AVANCE^III^ and AVANCE NEO 9.4 T wide bore NMR spectrometers driven by ParaVision 5.1 and 360 v3.0, respectively, and operating at a frequency of 400.21 MHz for ^1^H. Images were acquired using the Bruker microimaging unit Micro 2.5 with actively shielded gradient sets (1.5 T/m) and 25 mm quadrature resonators (Bruker). Pupae were carefully wrapped in foam rubber and fixed within the animal handling system, while imagoes were anaesthetized with 1.5% isoflurane using a home-build nose cone. This was easily tolerated by the moths, which awaked 1–2 min after removal of the inhalation anesthesia. Due to the vertical orientation of the MRI system, all animals were scanned in an upright position.

#### High resolution anatomical imaging

After acquisition of pilot scans, images were recorded in axial, sagittal and coronal orientation using rapid acquisition with relaxation enhancement (RARE) sequences with and without fat suppression. The following parameters were utilized: sagittal/coronal; 30 slices, slice thickness (ST) 0.5 mm, field of view (FOV) 32 × 16 mm^2^, matrix 1024 × 512, echo time (TE) 28.66 ms, repetition time (TR) 3500 ms, RARE factor 8, averages (NA) 2, acquisition time (TAcq) 3 min and 44 s; axial; 60 slices, ST 0.5 mm, FOV 16 × 16 mm^2^, matrix 512 × 512, TE 28.66 ms, TR 3988.13 ms, RARE factor 8, NA 2, TAcq 4 min and 15 s.

#### Determination of water and fat distribution

In order to calculate water and fat contents within the pupal body we made use of a middle slice acquired in coronal orientation with and without fat suppression. To produce essentially fat-only images, fat-suppressed data were subtracted in absolute intensity mode from non-suppressed data sets. For both data sets, we defined regions of interest (ROI) over the entire body surface of the pupae (n = 5 males and n = 5 females). Thereafter, mean fat and water signal intensities were calculated from the difference in signal intensity between the two measurements. In order to measure shifts in water distribution, we defined ROIs covering the head and thorax region in 5 defined middle slices per pupa and determined the mean water signal in fat suppressed images.

*For monitoring of active ventilation* a time-resolved RARE sequence was applied with TE 5.77 ms, TR 2500 ms, FOV 32 × 16 mm^2^, matrix 384 × 192, ST 0.75 mm, 60–120 repetitions, RARE factor 8, TAcq 60–120 min. Acquisition of T2 maps was carried out using a multi-spin-echo sequence (16 echoes, separated by a ΔTE of 4.6 ms; TR = 500 ms, FOV, 12 × 12 mm^2^; ST, 0.5 mm; matrix 256 × 256; NA, 4; TAcq of 5 min 22 s) and subsequent parametric analysis using the tools provided by ParaVision.

*For visualization of cardiac motion*, images were acquired in sagittal orientation adapted to the anatomical course of the aorta using a retrospectively gated fast low angle shot sequence (IntragateFLASH, Bruker) with a FA 10°, TE 1.69 ms, TR 6.42 ms, FOV 32 × 12 mm^2^, MS 340 × 128, ST 1 mm, NA 300, TAcq 7 min 21 s. For retrospective gating, an in parallel navigator slice (ST 3 mm) was placed over the entire pupa to monitor the required signals for artefact-free reconstruction^43^.

*MR angiography* (MRA) was carried out using a flow-compensated 2D time-of-flight (TOF) FLASH sequence (FA 80°, TE 2.54 ms, TR 10 ms, NA 3, TAcq 10 min 4 s, FOV 16 × 16 mm^2^, MS 192 × 192 zerofilled to 384 × 384). In z-direction the FOV was set to cover the entire pupa (140 overlapping slices with a slice thickness (ST) of 0.3 mm and an interslice distance of 0.18 mm resulting in a package extension of 25.32 mm). For 3D visualization, data were imported into Amira 4.0 (Mercury Computer Systems) and resampled to isotropic voxel size using a Lanczos filter. Afterwards, the surface of the pupa was visualized in semi-transparent greyscale and the signals from the flowing blood by texture-based volume rendering using the ‘glow’ colormap.

### Ethics approval

Unlike vertebrate research animals, working with insects does not require approval in Europe or the United States. Saturnia pavonia is widespread in Central Europe, common almost everywhere, and not an endangered species.

## Supplementary Information

Supplementary Information 1.

Supplementary Video 1.

Supplementary Video 2.

Supplementary Video 3.

Supplementary Video 4.

Supplementary Video 5.

Supplementary Information 2.

Supplementary Information 3.

Supplementary Information 4.

## Data Availability

All data supporting the findings of this study are available within the article and its supplementary material.
