# Supplementary material for: Dynamic monitoring of vital functions and tissue re-organization in Saturnia pavonia (Lepidoptera, Saturniidae) during final metamorphosis by non-invasive MRI
Source: Sci Rep. 2022 Jan 20;12:1105. doi: 10.1038/s41598-022-05092-3 (PMC8776771; doi:10.1038/s41598-022-05092-3)
Supplement: Supplementary file 1 — Supplementary Information 1. [file 41598_2022_5092_MOESM1_ESM.pdf]

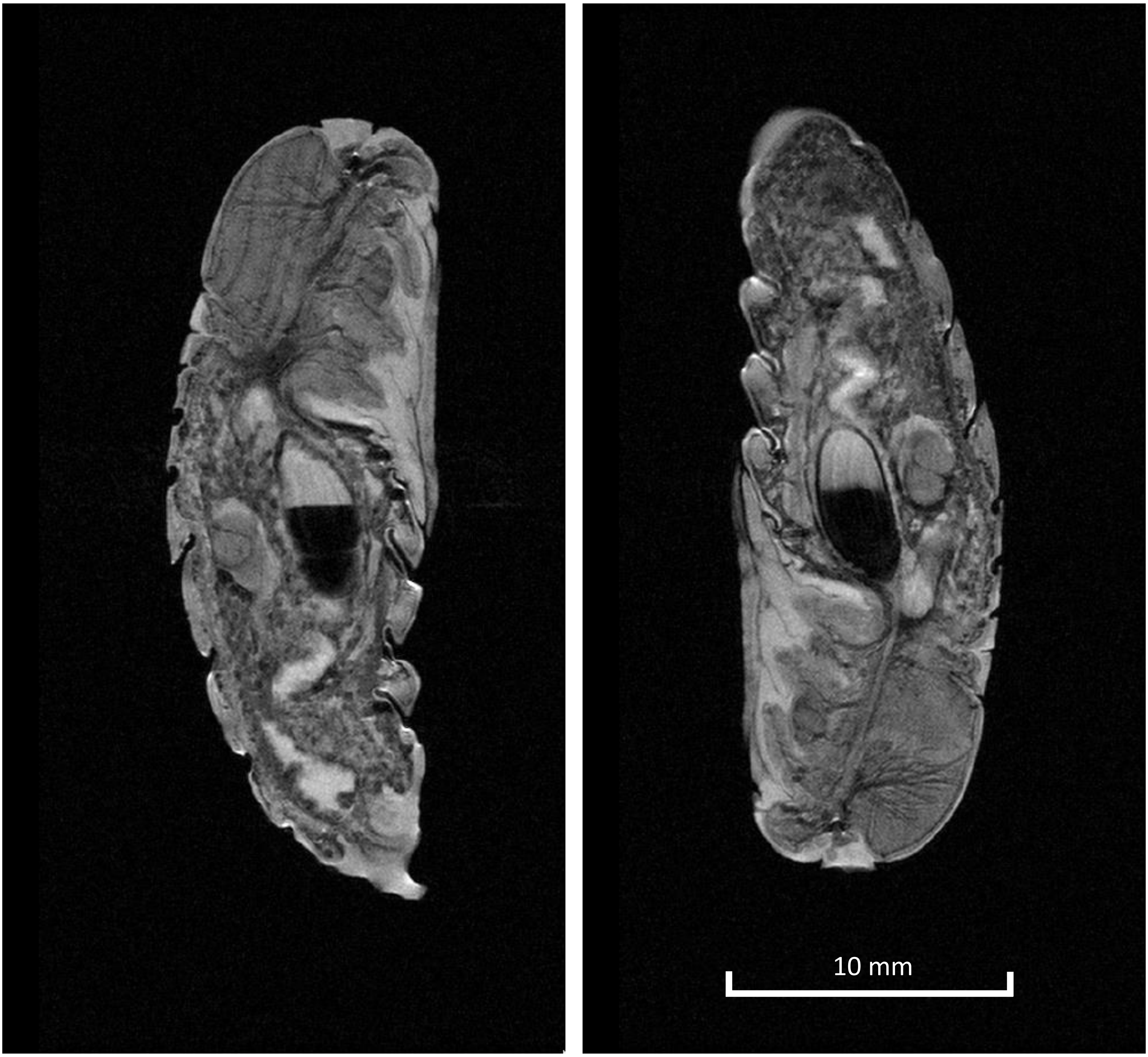

Supplemental figure 1: Effect of gravity on liquid and sediment in the gut of *Saturnia Pavonia* (coronal section of a male pupa).
