# Supplementary figures and images for: Dynamic monitoring of vital functions and tissue re-organization in Saturnia pavonia (Lepidoptera, Saturniidae) during final metamorphosis by non-invasive MRI
Source: Sci Rep. 2022 Jan 20;12:1105. doi: 10.1038/s41598-022-05092-3 (PMC8776771; doi:10.1038/s41598-022-05092-3)

20 mm (1-6)

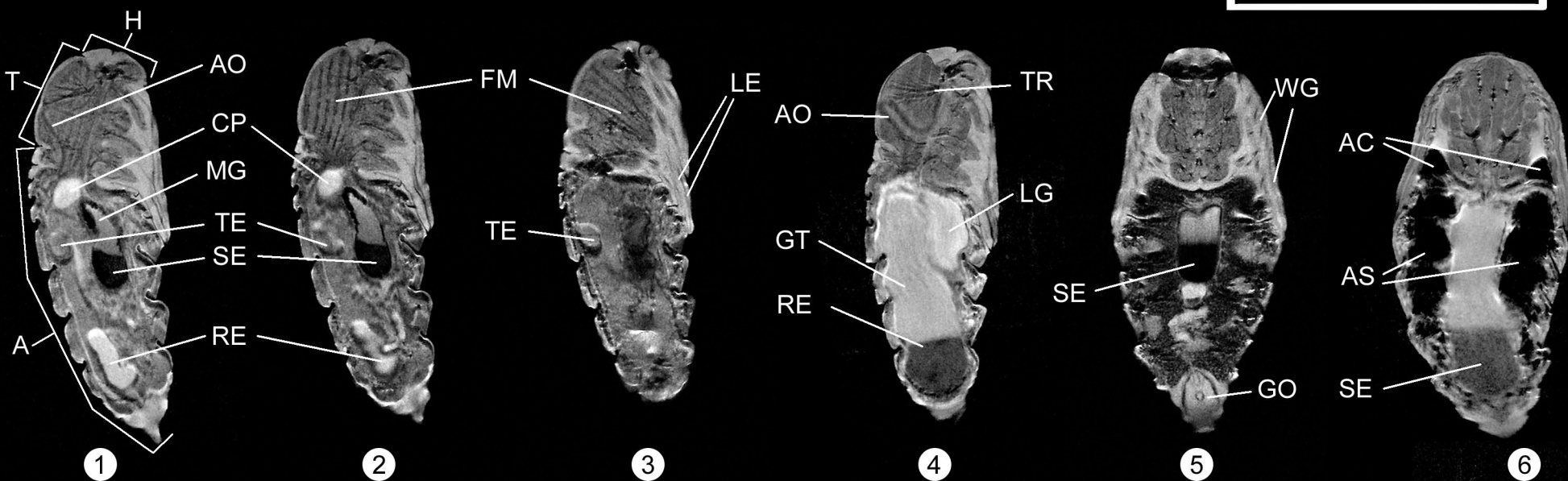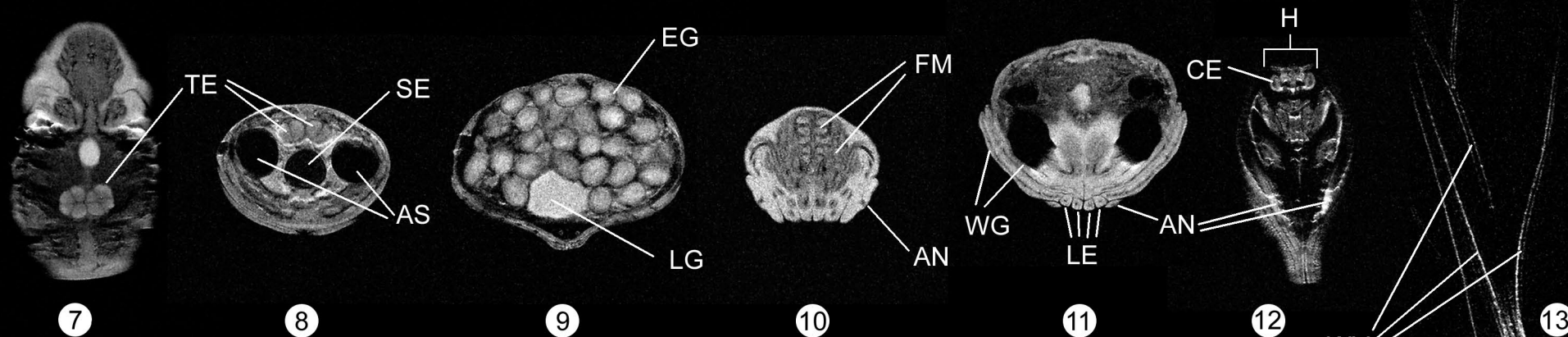

10 mm (9-13)

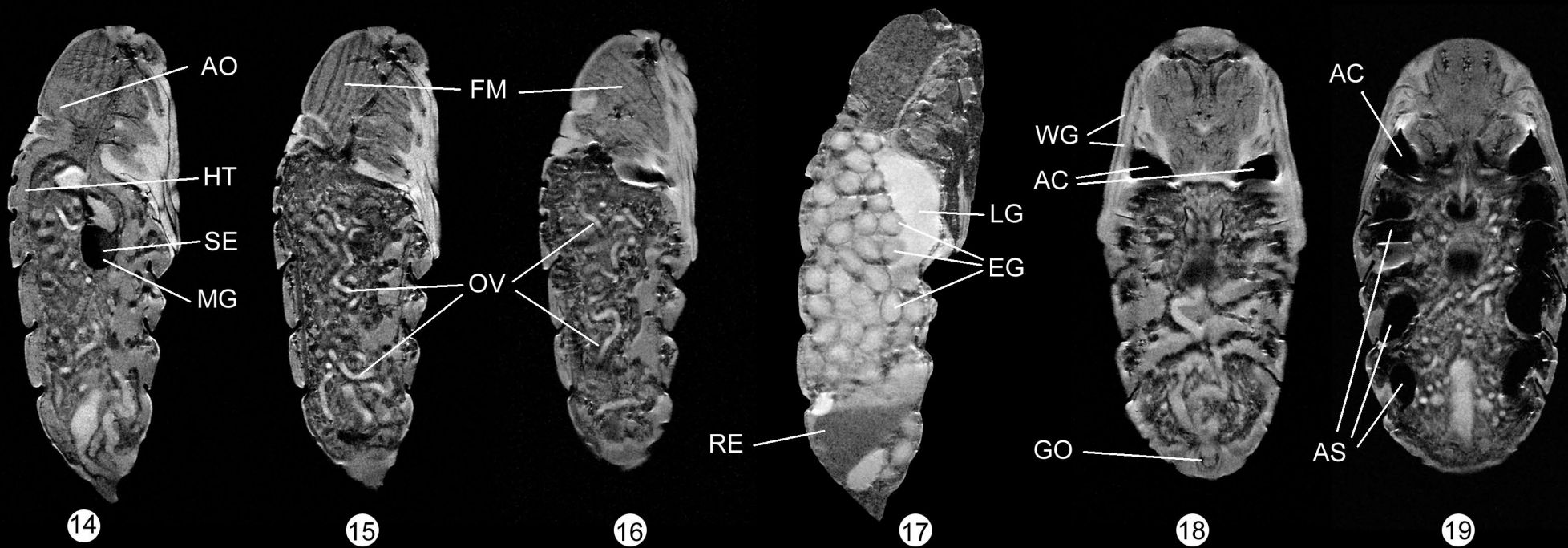

20 mm (14-19)

Supplement: Supplementary file 7 — Supplementary Information 2. [file 41598_2022_5092_MOESM7_ESM.pdf]
