# Supplementary material for: Dynamic monitoring of vital functions and tissue re-organization in Saturnia pavonia (Lepidoptera, Saturniidae) during final metamorphosis by non-invasive MRI
Source: Sci Rep. 2022 Jan 20;12:1105. doi: 10.1038/s41598-022-05092-3 (PMC8776771; doi:10.1038/s41598-022-05092-3)
Supplement: Supplementary file 8 — Supplementary Information 3. [file 41598_2022_5092_MOESM8_ESM.pdf]

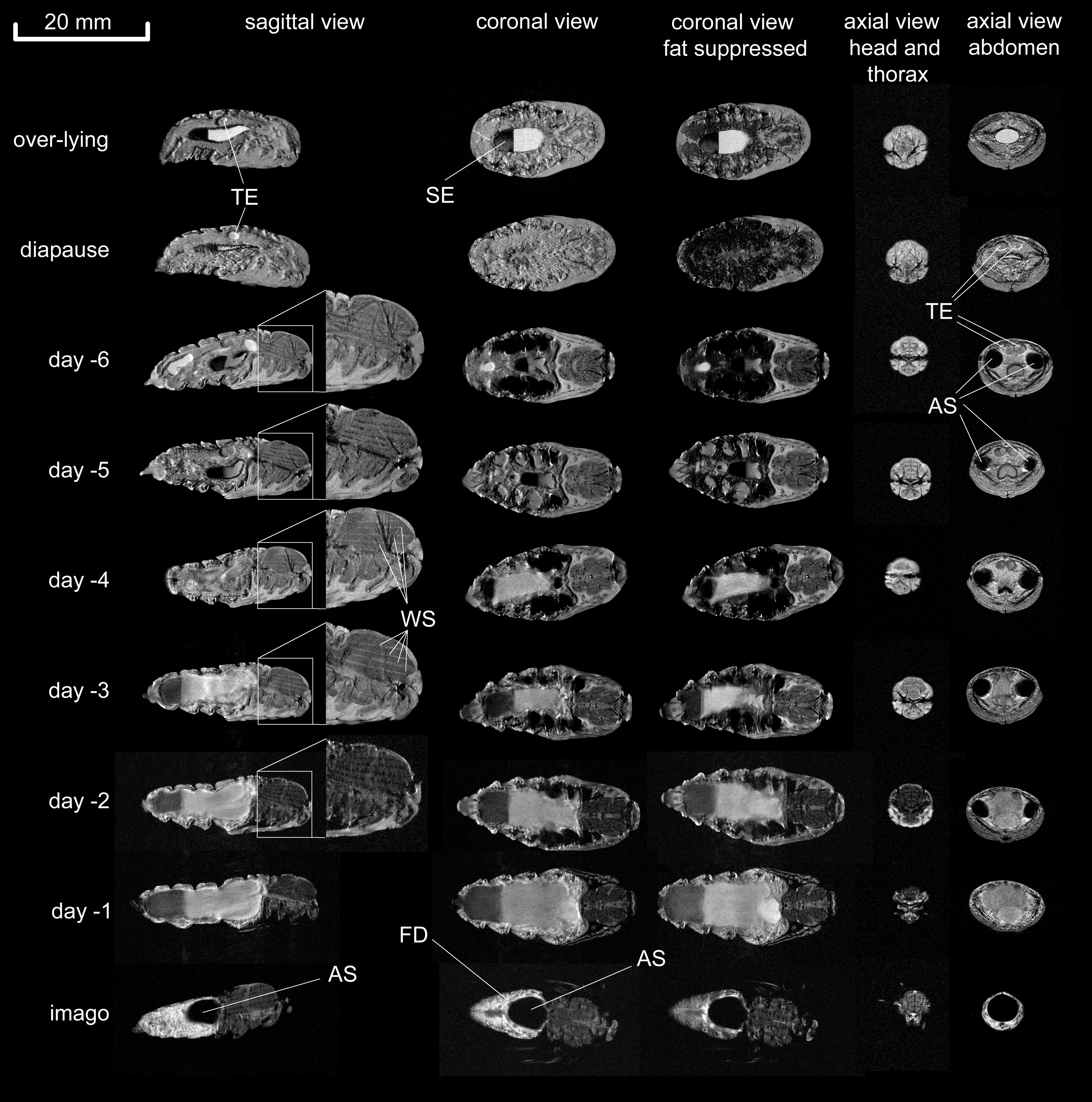

20 mm
sagittal view
coronal view
coronal view fat suppressed
axial view head and thorax
axial view abdomen
over-lying
diapause
day -6
day -5
day -4
day -3
day -2
day -1
imago
TE
SE
TE
AS
WS
FD
AS
AS
